# Inequality and income segregation in Brazilian cities: a nationwide analysis

**DOI:** 10.1007/s43545-022-00491-9

**Published:** 2022-09-10

**Authors:** José Firmino de Sousa Filho, Gervásio F. dos Santos, Roberto F. Silva Andrade, Aureliano S. Paiva, Anderson Freitas, Caio Porto Castro, Amélia A. de Lima Friche, Sharrelle Barber, Waleska T. Caiaffa, Maurício L. Barreto

**Affiliations:** 1 Center of Data and Knowledge Integration for Health (CIDACS), Salvador, Brazil; 2 School of Economics (PPGE), Federal University of Bahia, Salvador, Brazil; 3 Institute of Public Health (ISC), Federal University of Bahia, Salvador, Brazil; 4 Observatory for Urban Health in Belo Horizonte (OSUBH), Federal University of Minas Gerais, Belo Horizonte, Brazil; 5 Department of Epidemiology and Biostatistics, Drexel University Dornsife School of Public Health, Philadelphia, USA; 6 Institute of Physics, Federal University of Bahia, Salvador, Brazil

**Keywords:** Segregation, Income dissimilarity index, Urban inequality, Brazil

## Abstract

Residential segregation has brought significant challenges to cities worldwide and has important implications for health. This study aimed to assess income segregation in the 152 largest Brazilian cities in the SALURBAL Project. We identify specific socioeconomic characteristics related to residential segregation by income using the Brazilian demographic census of 2010 and calculated the income dissimilarity index (IDI) at the census tract level for each city, subsequently comparing it with Gini and other local socioeconomic variables. We evaluated our results’ robustness using a bootstrap correction to the IDI to examine the consequences of using different income cut-offs in substantial urban and regional inequalities. We identified a two minimum wage cut-off as the most appropriate. We found little evidence of upward bias in the calculation of the IDI regardless of the cut-off used. Among the ten most segregated cities, nine are in the Northeast region, with Brazil's highest income inequality and poverty. Our results indicate that the Gini index and poverty are the main variables associated with residential segregation.

**Supplementary Information:**

The online version contains supplementary material available at 10.1007/s43545-022-00491-9.

## Introduction

The phenomenon of the expansion of cities and the transformation of urban areas has led to the creation of various forms of spatial territories. Central and peripheral areas are objects of theoretical and empirical studies worldwide. Since then, socioeconomic configurations have emerged from dynamic city centers with multiple and often conflicting features. In this sense, residential segregation and inequality arise due to a series of actions that can be subjective and individual or material due to a superstructure of discrimination (Iceland et al. 2002; Reardon and Bischoff 2011; Schelling 1971; White 1983).

The dimensions of segregation include evenness, exposure, concentration, clustering, and centralization (Acevedo-Garcia et al. 2003; Garcia-López and Moreno-Monroy 2018; Massey and Denton 1988). The heterogeneity of services, infrastructure, access, or lack of resources contained in each city neighborhood differentially impacts residents’ income conditions and affects the segregation process. Segregation and inequality are severe problems observed worldwide (United Nations 2020). In Latin American countries, and Brazil, in particular, the situation is often especially stark (Ferreira and Ravallion 2008).

Understanding income segregation in Brazil is essential given the negative economic and social consequences for households in more vulnerable urban areas. Low-income groups living in segregated cities have notably fewer educational opportunities, access to mobility, and lower wages than their peers in more integrated areas, resulting in severe economic disadvantage. In this way, we can say that the progress of studies related to the income dissimilarity index brings relevant contributions to the scientific perspective and to elaborating public policies in Brazilian cities. Spatial patterns of income distribution have shaped the forms and construction of cities since the differences between low- and high-income groups demand goods and services of different complexities, which interferes with social networks and interactions between individuals (Marques 2015).

Henceforth, we used the dissimilarity index to examine the association between income segregation and inequality as major factors for promoting equity and social development in large Brazilian urban centers. The Income Dissimilarity Index (IDI), initially proposed by Duncan and Duncan (1955), allows a spatial view of the population that would need to be “moved” within a city to make it homogeneous in terms of income. It is a measure of average segregation that indicates how far the poorest are from the city’s average income (Massey and Danton 1988; Iceland et al. 2002). To define an income cut-off at the national level, we initially tested different low-income minority groups: households that earn 0 to ½, 0 to 1, 0 to 2, and 0 until three minimum wages.^1^ Subsequently, we obtained the IDI based on the income data of all 164,109 census tracts of 152 Brazilian cities as defined by the SALURBAL project (Diez-Roux et al. 2019; Quistberg et al. 2019).

The *Salud Urbana en Latin América* (SALURBAL) or Urban Health in Latin America project is an initiative that aims to support urban policies that promote health and health equity in cities of the region (Diez-Roux et al. 2019). In addition, it addresses a comprehensive scope of research related to urban environments, sustainability, and social equity, such as poverty, income inequality, housing conditions, education, and employment. Therefore, this work is concisely inserted into the project as it analyzes how socioeconomic factors are related and provides a consistent basis for future policy research for global measures aiming to reduce inequalities.

This paper seeks to answer three important research questions: (i) Is there a bias in the IDI to make the indicator inconsistent? (ii) What is the best income cut-off for shares of low-income households to calculate IDI? (iii) how is the IDI associated with other socioeconomic indicators in a context of high regional inequality?

Our first contribution is a quantitative analysis of the IDI based on the concern expressed by Taeuber and Taeuber (1976). They pointed to the possibility of an upward bias in the dissimilarity index measured by race in US cities where the representative group of black people would be much smaller than the group of white people. In these cases, the dissimilarity index had a high value. Furthermore, the larger the subareas, the less likely there was an exact match between the number of minority households and the total number of households in any subareas. Henceforth, other studies advanced on analyzing the dissimilarity index based on race and outcomes in education, income, employment, health, and others. Sophisticated methods and tools were used to attest to the consistency of the dissimilarity index, as well as other indices like entropy, where isolation can be easily calculated to measure residential segregation (Lee et al. 2015; Tivadar 2019; Yalonetzky 2012).

Royuela and Vargas (2010) and Mazza (2017) state that the dissimilarity index remains the most commonly used measure of residential segregation. We add to this rich literature the analysis of this indicator at different income levels in a context of urban areas with evident inequalities, as is the case of the largest Brazilian cities. Using the bootstrap method, we observed no relevant bias embedded in the income-based dissimilarity index through sensitivity analyses (Allen et al. 2015; Tivadar 2019). We further highlight that, to the best of our knowledge, no study has yet addressed this scope for the income-based dissimilarity index. However, this topic can easily be replicated in other countries, regions, or cities (Tivadar 2019).

Torres (2006) calculated the dissimilarity index for the metropolitan region of the city of São Paulo based on the 1991 and 2000 censuses. The author compared levels of segregation between census tracts and larger spatial areas such as districts and concluded that the dissimilarity index for census tracts has a better consistency. In addition, it was pointed to an income cut-off of 3 minimum wages to indicate the low-income group since it represented 39.0% of the population in 1991 and 31.4% in the 2000 census.

Here, we found that the cut-off of 2 minimum wages is adequate to define a low-income minority group at the national level in Brazil since it represents 33.8% of the sample according to the census of 2010. It means that the percentage of the low-income group is a major factor in the analysis of residential segregation measured by income. Therefore, in the context of great regional income inequality, the spatial distribution of the low-income minority is essential to measure the heterogeneity within a city and efficiently capture the level of segregation. The heterogeneity we refer to means reaching a low-income minority group capable of adequately representing intra-urban and regional inequality patterns. Finally, using linear regression models, we find that the Gini index and poverty are the main variables associated with residential segregation, measured by the IDI.

Our work contributes in multiple dimensions to the socioeconomic field of studies on segregation. First, the evaluation of the IDI by income levels in one of the unequal countries in the world is something to be taken into account since the large Brazilian cities concentrate more than 85% of the country’s population. Defining a cut-off for low-income minority groups is necessary to have a consistent indicator in quantitative and qualitative analyses, as the interpretation of the IDI can be adapted in different economic, sociological, geographic, and public health contexts, among others. Regarding the tools and means of measurement for indicators of segregation by income, race, education, or others, the application of random sampling tests developed by Tivadar (2019) showed a robust effect. It also was pointed out by other studies such as Allen (2015), Mazza and Punzo (2015), and Mazza (2017) as an effective alternative to correct possible upward bias in the dissimilarity index. Results show no relevant changes in the associations between the IDI and other socioeconomic variables, which was expected. Even so, we emphasize that future research needs to advance in new techniques to rectify and consolidate means and tools of measuring segregation and inequality indicators.

## Segregation in the Brazilian context

We can define income segregation as the uneven classification of households according to their income level within the urban space (Reardon and Bischoff 2011). The urban spatial structure is an essential determinant for allocating people, industries, jobs, and other factors, which impact the distribution of resources for developing public policy strategies involving sanitation, education, and public health, among others (Garcia-López and Moreno-Monroy 2018). Hence, segregation creates barriers and limits opportunities for access to basic goods and services for households’ personal and collective development.

Residential segregation accentuates social and economic inequalities and reveals an unequal social structure. Although segregation can sometimes be barely presented as fractioned spatial arrangements, it is an important dimension of social inequality that impedes social mobility since groups are distinguished concerning social isolation or exclusion. It is reflected in cities where the economically most vulnerable people have significant barriers to getting to work, young people going to school, and other dimensions of social interactions and engagement.

Telles (1995) assessed the effect of structural factors, such as urbanization and industrialization, on income segregation in the most prominent Brazilian metropolitan regions. According to the author, the processes of urbanization and industrialization are independent and have different effects on income segregation. In the Brazilian case, industrialization decreased segregation, while urbanization had the opposite effect. Given the substantial inequality in Brazil’s industrialized urban areas, new forms of industrialization can reduce socioeconomic inequalities and income segregation. Regarding regional issues, the unequal metropolitan regions were those of the Northeast region (except for Brasília, in the Center-West). Salvador, Recife, João Pessoa, and Teresina were configured as highly segregated regions. However, the metropolitan region of Brasília has become the most segregated. Despite the planned urban form, the real estate market and unforeseen population growth soon surpassed the city limits, creating unplanned “satellite cities” formed mainly by settlements and slums.

In the field of the regional economy, studies such as Akita (2003), Elbers et al. (2003), Miranti et al. (2015), Trendle (2005), Tarozzi and Deaton (2009) used statistical and spatial methods to estimate income inequalities, poverty, demographics, Gini, market composition workplace, educational issues and, racial segregation. The literature argues that policies to reduce regional inequalities, income, and greater social welfare are beneficial.

Torres (2006) evaluated the importance of discussions on residential segregation concerning issues related to housing, sanitation, and other public policies in the city of São Paulo. Using the 1991 and 2000 Brazilian demographic censuses, the author calculated the dissimilarity index for different income cut-offs. An increase in segregation in the city was observed, especially when comparing households that earn up to 3 minimum wages and households that earn more than 20 minimum wages. It showed a trend toward social distancing those with greater purchasing power from more expansive society spaces. This movement has been widely observed in other large cities in Latin America, creating and expanding “gated communities” (Sabatini et al. 2001; Peters and Skop 2007; Figueroa et al. 2021). For the author, segregation is a phenomenon that has consequences in a broad sense, but it can be mitigated with income distribution and housing policies. However, the rapid urbanization of cities in Latin America makes policies that alleviate residential segregation difficult. The lack of funding for public works, increased violence, and social degradation of marginalized individuals.

Marques (2015) theoretically analyzed the relationship between urban poverty, residential segregation, and social connections in two Brazilian cities, São Paulo and Salvador. The study showed significant socioeconomic heterogeneity between social strata, especially among middle-class individuals. In addition, connections and sociability among individuals were associated with good housing conditions, employment, and income level. Individuals who live in worse social conditions are more segregated and have few connections with other groups, losing access to public services and the “structure of opportunity” or “sources of well-being” (Mustered et al. 2006; Kaztman 2003). Finally, segregation tends to be more restrictive and limits social connections for poorer populations in more unequal cities such as Salvador. For middle-class individuals or households, living in São Paulo or Salvador makes no difference to their connections or sociability.

Santos et al. (2021) calculated income segregation for Brazilian cities and highlighted how it affects homicide patterns in large urban centers. According to the authors, segregation is further aggravated by high regional inequalities in Brazil, and under these circumstances, there is a discriminatory social structure that involves the spatialization of inequality and poverty. Thereby affecting the collective health of individuals and households that find themselves in a situation of socioeconomic vulnerability.

Despite income gains in the first decade of the XXI century, economic growth in Brazil was not reflected in better income distribution (IBGE 2017). Segregation remains evident in large urban centers. Still, residential integration may not be enough to close the income gap between social groups. Policies aimed at education, permanent income gains for the lower classes, and employment opportunities must continue to facilitate forms of integration and social connections.

## Data and methods

### Data

We used the sample of the 152 Brazilian cities included in the *Salud Urbana en Latin América* (SALURBAL) project comprising urban agglomerations with more than 100,000 inhabitants in 2010. The urban agglomerations (L1ADs) comprise 422 sub-city units (municipalities). They can be defined as a single administrative unit (e.g., municipality) or a combination of adjacent administrative units (e.g., several municipalities) that are part of an urban extension determined from satellite imagery (Quistberg et al. 2019).

Income data by census tract were collected by the Brazilian Bureau of statistics (IBGE, Portuguese acronym) for 2010. In Brazil, data at the census tract level are collected every ten years. The 2020 demographic census has been postponed to 2022 because of the COVID-19 pandemic. The income segregation index is based on the number of private households under certain minimum wage thresholds. For the dissimilarity index, which compares two social groups, the index was calculated for the following pairs: households earning from 0 to ½ minimum wage vs. the total (households with or without income); households earning 0 to 1 wage vs. the total; households earning 0 to 2 wages vs. the total and, finally, households earning 0 to 3 wages vs. the total. The IBGE did not compute households that did not report income for this category (IBGE 2011).

The socioeconomic variables examined with IDI are shown in Table 1. SEI is an indicator that considers the level of education (proportion of the population aged 25 or older who completed primary education or above), access to water, sanitation, and housing conditions based on the number of households overcrowding. The overcrowding indicator was reverse coded so that higher values of all measures indicate a better social environment. Finally, the four measures were summed and divided by 4, assuming equal weights for each (Bilal 2021). The SEI index and other covariates were transformed into Z-scores.

**Table 1:** Variables used in the regression, all data for 2010

Variables	Definition
Population	Projected population
Gini index	Income inequality based on the household total income
GDP per capita	Nominal GDP (UU$ dollars)/Population
Unemployment	The unemployment rate among the total population 15 years or above in the labor force
Poverty rate	The proportion of the population living in households with household income below the national income poverty line
Social Environment Index (SEI)	Education / water access / sanitation / overcrowding (reverse coded). Indices summed and divided by 4 assuming equal weights for all four measures

Source: SALURBAL Project

Three models were estimated, taking the IDI as the exposure variable. In model 1, the Gini index was used as the outcome and the SEI in the second. In the third model, we included all other covariates.

### Definition of the dissimilarity index

The allocation of individuals within a space can be purely random or reflect the influence of economic, social, and environmental determinants. In general, the systematic allocation process is influenced by the preferences or restrictions of individuals and occupied areas.

Allen et al. (2015), Mazza and Punzo (2015), and Mazza (2017) offer statistical tools capable of measuring the distribution of individual allocations in space through the dissimilarity index. All specifications described here are based on the methodology developed by these authors. Hereafter, we assume that $j=1, \dots , J$, units are nested within a given urban area. Individuals may or may not have measurable characteristics on a dichotomous scale, $c=0, 1$. The number of individuals with status $c$ in the urban area is denoted ${n}^{c}$. Individuals are allocated, ${n}_{j}^{c}$ individuals in unit $j$ having status $c$, and the total number of individuals in unit $j$ is represented by ${n}_{j}= {n}_{j}^{1}+{n}_{j}^{0}$.

Then, we can use the dissimilarity index $D$ for a given region as follows:

2.2.1 $$ \begin{gathered} D = ^\circ \frac{1}{2}\sum\limits_{j = 1}^{J} {\left| {\frac{{n_{j}^{1} }}{{n^{1} }} - \frac{{n_{j}^{0} }}{{n^{0} }}} \right|} \hfill \\ \hfill \\ \end{gathered}. $$

The allocation is given by a set of probabilities ${p}_{j}^{c}$ that assigns individual $i$ for unit $j$ according to individual status $c$:

2.2.2 $$ p_{j}^{a} \equiv P\left( {unit = j{|}c = a} \right),\, j = 1, \ldots , J;\,\,a = 0,1. $$

Systematic segregation is present when there is $j$ such that ${p}_{j}^{1}\ne {p}_{j}^{0}$. The relationship between $D$ and the underlying allocation probabilities is denoted by the fraction $\frac{{n}_{j}^{c}}{{n}^{c}}$, $c$ = 0,1. Since ${\widehat{p}}_{j}^{c}= \frac{{n}_{j}^{c}}{{n}^{c}}$, therefore, $D$ is only half of $\sum_{j=1}^{J}|{\widehat{p}}_{j}^{1}-{\widehat{p}}_{j}^{0}|$. The objective is to recognize the dissimilarity index as an estimator for the population quantity to allocate individuals independently. Thus, the population of a region with an individual number $n$, proportionally $p= {n}^{1}/n$ with status $c=1$ is allocated in $J$ units according to the ${p}_{j}^{c}$ probability. The probability function captures all allocations, and the results are the allocations ${n}_{j}={n}_{j}^{1}+{n}_{j}^{0}$ determined by a stochastic allocation, and the sizes of the units are given by:

2.2.3 $$ E\left[ {n_{j} } \right] = n^{1} p_{j}^{1} + n^{0} p_{j}^{0}. $$

Therefore, we can rewrite $D$ as follows:

2.2.4 $$ D_{pop} = \frac{1}{2}\mathop \sum \limits_{j = 1}^{J} \left| {p_{j}^{1} - p_{j}^{0} } \right|, $$

${D}_{pop}=0$ if and only if ${p}_{j}^{1}={p}_{j}^{0} \forall j.$

Maximum likelihood functions can estimate the conditional probabilities as independent allocations of multinomial distributions, and the function’s log-likelihood can be described as:

2.2.5 $$ \log L = \log \left( {\frac{{n^{1} !}}{{n_{1}^{1} ! \ldots n_{J}^{1} !}}} \right) + \log \left( {\frac{{n^{0} !}}{{n_{1}^{0} ! \ldots n_{J}^{0} !}}} \right) + \mathop \sum \limits_{j = 1}^{J} n_{j}^{1} \log (p_{j}^{1} ) + \mathop \sum \limits_{j = 1}^{J} n_{j}^{0} \log (p_{j}^{0} ). $$

The maximum likelihood estimator is given by ${\widehat{p}}_{j}^{1}=\frac{{n}_{j}^{1}}{{n}^{1}}$ e ${\widehat{p}}_{j}^{0}=\frac{{n}_{j}^{0}}{{n}^{0}}$, $j=1,\dots , J.$ expressed by the product of two independent multinomial distributions, one for $c=0$ and one for $c=1$:

2.2.6 $$ P\left( {n_{1}^{0} , \ldots , n_{j}^{0} , n_{1}^{1} , \ldots , n_{j}^{1} ;p_{1}^{0} , \ldots , p_{j}^{0} ,p_{1}^{1} , \ldots ,p_{j}^{1} ,n^{0} ,n^{1} } \right) = \mathop \prod \limits_{j = 1}^{J} \mathop \prod \limits_{c = 0}^{1} n^{c} ! \frac{{\left( {p_{j}^{c} } \right)^{{n_{j}^{c} }} }}{{n_{j}^{c} !}}. $$

When we assume that the size of units ${n}_{j}$ is fixed, we apply another model and additionally assume that the size of population $n$ and the minority proportion $p$ are also fixed. The allocation can be given by conditioned probabilities and ${D}_{pop}$ is written as follows:

2.2.7 $$ \begin{gathered} D_{pop} = \frac{1}{2}\mathop \sum \limits_{j = 1}^{J} p \left( {unit = j} \right)\left| {\frac{{P\left( {c = 1|unit = j} \right)}}{{P\left( {c = 1} \right)}} - \frac{{1 - P\left( {c = 1|unit = j} \right)}}{{1 - P\left( {C = 1} \right)}}} \right| \hfill \\ \quad \quad = \frac{1}{2}\mathop \sum \limits_{j = 1}^{J} \frac{{n_{j} }}{n}\left| {\frac{{P\left( {c = 1|unit = j} \right)}}{{P\left( {c = 1} \right)}} - \frac{{1 - P\left( {c = 1|unit = j} \right)}}{{1 - P\left( {C = 1} \right)}}} \right| \hfill \\ \end{gathered}. $$

Therefore, with a complete population or with a random sample, $D$ will remain an estimator of ${D}_{pop}$, both in cases of random effects or units of fixed sizes. This statistical distribution allows us to demonstrate whether or not there is bias in the income dissimilarity index, as described below.

### Measuring the presence of bias in the index

The bias can arise when the area population is small or the minority group proportion is very low. Consequently, the index is affected by differences in the proportion of the minority in the population and the size of the areal unit of analysis, making it difficult to compare the indices across cities. Fosset (2017) presents a series of “rule-of-thumb” practices to minimize the problem of bias, among which we highlight: (i) the use of census tracts and (ii) focus on the comparison between broader minorities and the rest of the population versus more finely categorized groups.

We followed these recommendations by working with census tract level data and explored the bias generated by different income cut-offs. In order to evaluate bias embedded in the IDI, we use randomization models with bootstrap. For that, we take as reference Efron (1979) in which for an estimator $\widehat{\theta }=s(x)$ the bootstrap bias estimator is defined as ${bias}_{\widehat{F}}$:

2.3.1 $$ bias_{F} = bias_{F} \left( {\hat{\theta },\theta } \right) = E_{F} \left[ {s\left( x \right)} \right] - t\left( F \right), $$

**Fig. 1:**
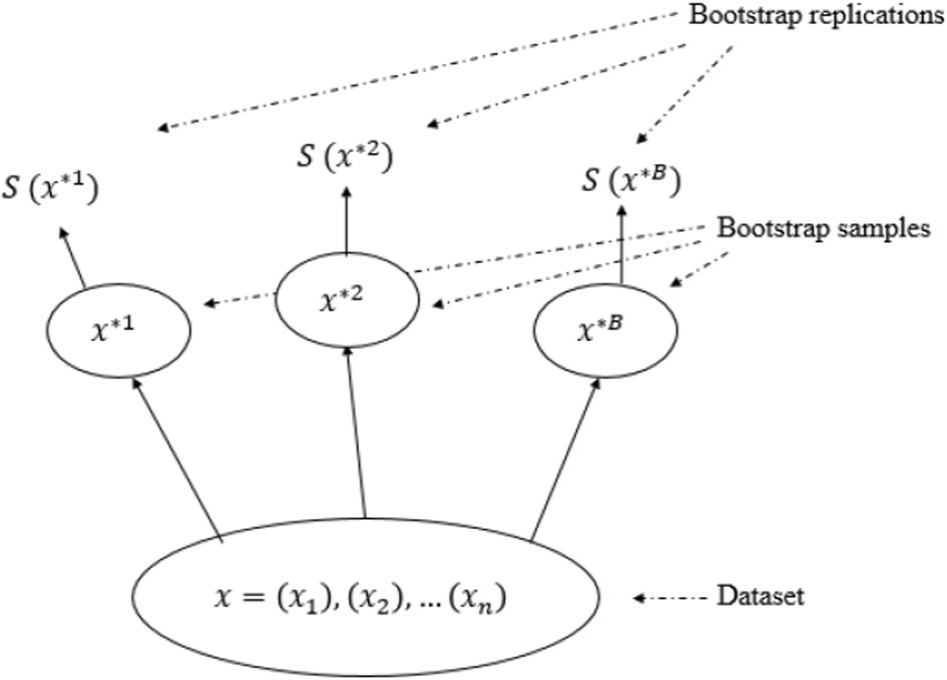
Illustration of bootstrap replications. Source: Efron, 1979

2.3.2 $$ bias_{{\hat{F}}} = E_{{\hat{F}}} \left[ {s\left( {x^{*} } \right)} \right] - t\left( {\hat{F}} \right), $$

where $t\left(\widehat{F}\right)$ is the estimator of $\theta$ is different from $\widehat{\theta }=s\left({x}^{*}\right)$. ${bias}_{\widehat{F}}$ is the plug-in estimator of ${bias}_{F}$, and $\widehat{\theta }$ may or may not be the $\theta$. Efron (1979) also demonstrates the ideal bootstrap estimator through simulations by Monte Carlo, in which independent bootstrap samples $\left({x}^{*1}\right), \left({x}^{*2}\right), \dots s\left({x}^{*B}\right)$, are generated, as in Fig. 1, calculating the bootstrap replications ${\widehat{\theta }}^{*}\left(b\right)=s\left({x}^{*b}\right)$ and approximating the expected value ${E}_{\widehat{F}} \left[s\left({x}^{*}\right)\right]$ by the mean

2.3.3 $$ \hat{\theta }^{*} \left( . \right) = \mathop \sum \limits_{b = 1}^{B} \frac{{\hat{\theta }^{*} \left( b \right)}}{B} = \mathop \sum \limits_{b = 1}^{B} \frac{{s\left( {x^{*b} } \right)}}{B}. $$

Bootstrap samples are generated from the original dataset. Each bootstrap sample has $n$ elements, generated by sampling with replacement $n$ times from the original dataset. Bootstrap replicates $s \left({x}^{*1}\right), \left({x}^{*2}\right), \dots s\left({x}^{*B}\right)$ are obtained by calculating the value of the statistic $s (x)$ on each bootstrap sample. Finally, the standard deviation of the values $\left({x}^{*1}\right), \left({x}^{*2}\right), \dots s\left({x}^{*B}\right)$ is our estimate of the standard error of $s (x)$.

The bootstrap bias estimator based on $B$ replications ${\widehat{bias}}_{B}$, is (3.3.2) with ${\widehat{\theta }}^{*}\left(.\right)$ replaced by ${E}_{\widehat{F}} \left[s\left({x}^{*}\right)\right]$:

2.3.4 $$ \widehat{bias}_{B} = \hat{\theta }^{*} \left( . \right) - t\left( {\hat{F}} \right). $$

Concerns about the bias embedded in the dissimilarity index are not recent. Carrington and Troske (1997) developed indices of systematic segregation that measure the distance of randomness samples instead of the distance of uniformity. The use of randomness in the allocation implies a substantial unevenness caused by small minority shares regarding the whole or census tracts that are not representative of income heterogeneity between households. It reinforces the need to check for bias in the indicator when examining associations with other socioeconomic, health, environmental, and built factors.

### Associations between the dissimilarity index and other socioeconomic variables

The linear regression method is a classic technique of statistical mathematics and an essential part of modern econometrics theory (Rao and Toutenburg 1995; Wooldridge 2019). We took a generalized and multivariable linear equation and ran three models for associations between the dissimilarity index and socioeconomic variables. In the first model, the outcome of interest is the Gini index traditionally used to measure income inequality between households, cities, or regions. In the second model, we use the social environment index as the outcome, critical aggregating characteristics of a city’s social development. Finally, in the third model, we introduce the entire set of covariates. Traditional tests to assess the robustness of the models were performed, such as the Breusch-Pagan/Cook-Weisberg test for heteroscedasticity, White's general test statistic, and the Durbin-Watson test. In all cases, tests stated that the OLS models were robust.

## Results and discussion

Table 2 provides an overview of our data. We stratified the sample of studied cities by population quartiles to observe the patterns of the different indicators according to the size of the city population. Both the dissimilarity and Gini indices increase with the population size. Cities in the second quartile of the population have a higher GDP *per capita* due to an accentuated growth in medium-sized cities in Brazil (Bolay 2020; Henderson 1997; Mata et al. 2005). This finding can also be explained by the high cost of living in the country's major metropolitan centers. It is reflected in monetary matters and related to households' quality of life and leisure in large cities.

**Table 2:** Descriptive statistics by city population quartiles

Variables	Q1*(N* = *38)* Mean (SD)	Q2 *(N* = *38)* Mean (SD)	Q2 *(N* = *38)* Mean (SD)	Q4 *(N* = *38)* Mean (SD)
Dissimilarity index	0.25 (0.04)	0.25 (0.04)	0.26 (0.04)	0.30 (0.04)
Gini	0.53 (0.03)	0.53 (0.02)	0.54 (0.03)	0.60 (0.04)
GDP *per capita (US$)*	14.2 (9.1)	18.5 (12.8)	16.5 (9.6)	17.3 (7.0)
Unemployment	0.09 (0.03)	0.08 (0.03)	0.08 (0.02)	0.09 (0.02)
Poverty rate	0.26 (0.15)	0.24 (0.12)	0.25 (0.13)	0.24 (0.11)
Social Environment Index (SEI)	0.07 (0.39)	0.02 (0.34)	0.09 (0.44)	0.19 (0.36)

Unemployment and poverty did not differ much between cities according to population size. However, the social environment index points out that larger cities can provide more services to improve the well-being of households.

**Fig. 2:**
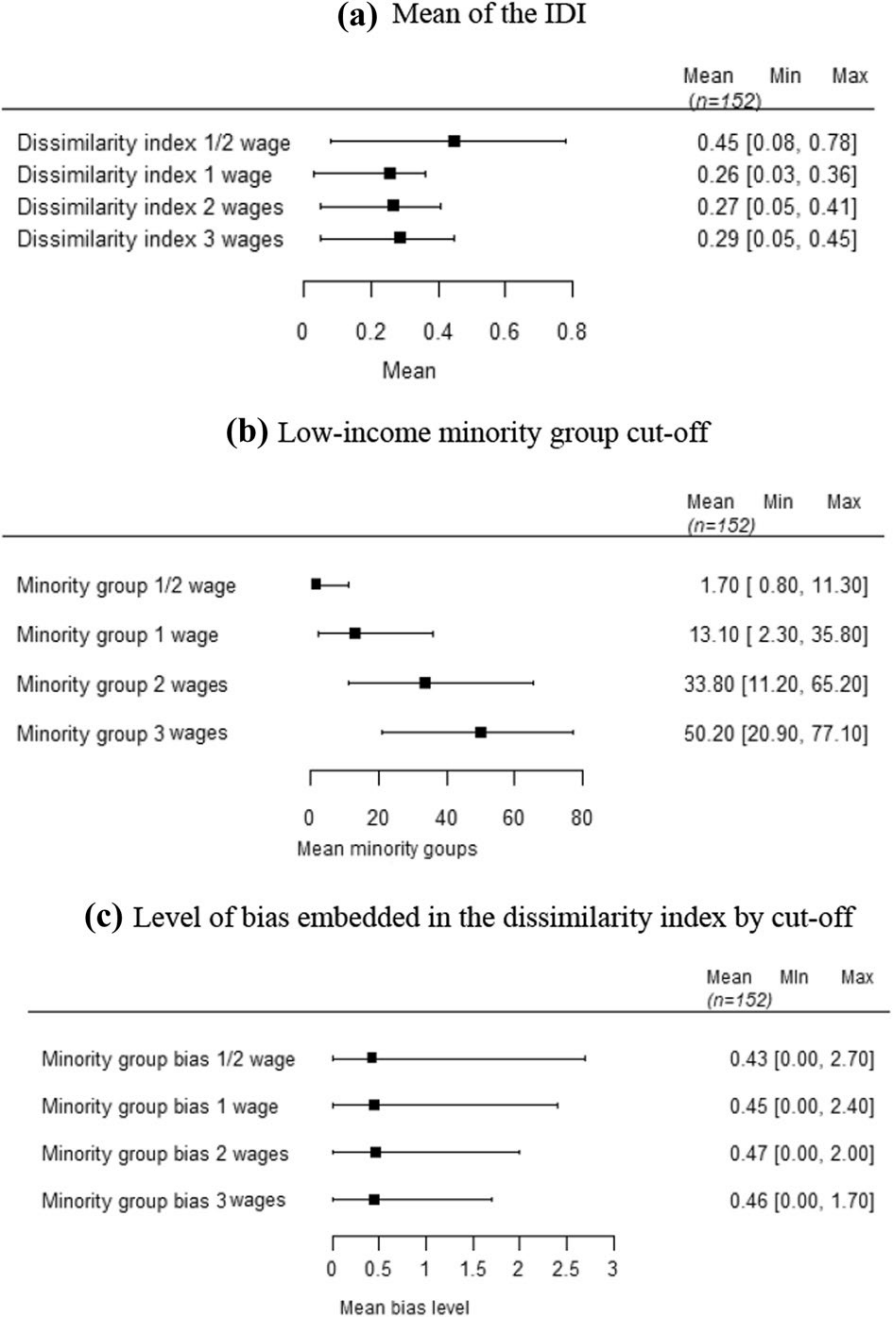
Descriptive statistics for the indices. Source: Research results

As shown in Fig. 2, the cut-offs analyzed for the 152 cities presented different average, maximum, and minimum levels. The mean income segregation of the ½ minimum wage cut-off was 45%. However, the proportion of households in the minority low-income group is tiny, with only 1.7% of Brazilian households Fig. 2b. Therefore, it is not reasonable to work with this cut-off.

Based on this same criterion, the indicator of 1 minimum wage also represents a small proportion of households; only 13.1% are in the low-income minority group. Therefore, cut-offs of 2 and 3 minimum wages present better-distributed samples and have average income segregation of 27% and 29%, respectively. However, the low-income cut-off related to households earning up to 3 minimum wages exceeds 50% of the sample, so this group cannot be used as a minority group.

The level of bias found in the IDI was low and did not appear to be dependent on the cut-off level used, as shown in Fig. 2c. The Brazilian bureau of statistics defines census tracts as a continuous cadastral control unit. Every sector must be fully contained in an urban or rural area, and its dimension in numbers of households and establishments must allow the enumerator to carry out the work within a specified period. The number of households and households in the census tracts is similar regardless of the city (IBGE 2020), which also explains the low level of bias. On average, for all income groups analyzed, the bias remained below 0.5%.

**Fig. 3:**
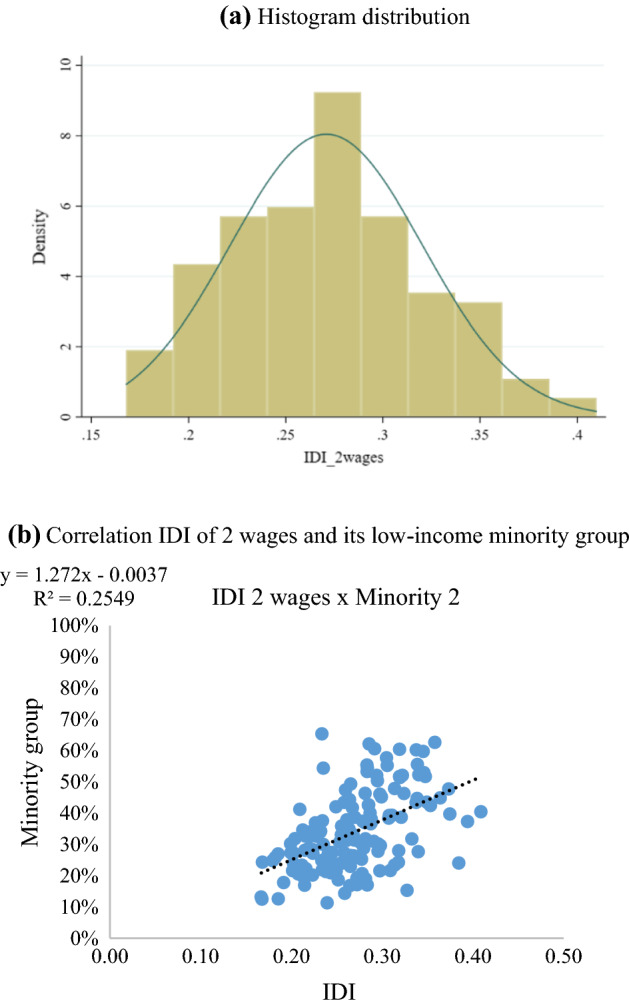
Statistical distribution for the income dissimilarity index. Source: Research results

Figure 3a shows the histogram for the distribution of the percent of households earning up to two minimum wages across cities. As the level of bias, specifically for the Brazilian case, was not dependent on the sample mean in any case, we can use the indicator with or without correction. Therefore, we use the indicator calculated initially by the SALURBAL Project. Figure 3b presents the distribution of 2 minimum wage IDI and cities' percentage of minority low-income groups. The correlation between both measures was approximately 0.25.

In subsequent analyses, we used the two wages cut-off to look for associations between income segregation indicators and other socioeconomic variables.

### Spatial distribution of indicators

**Fig. 4:**
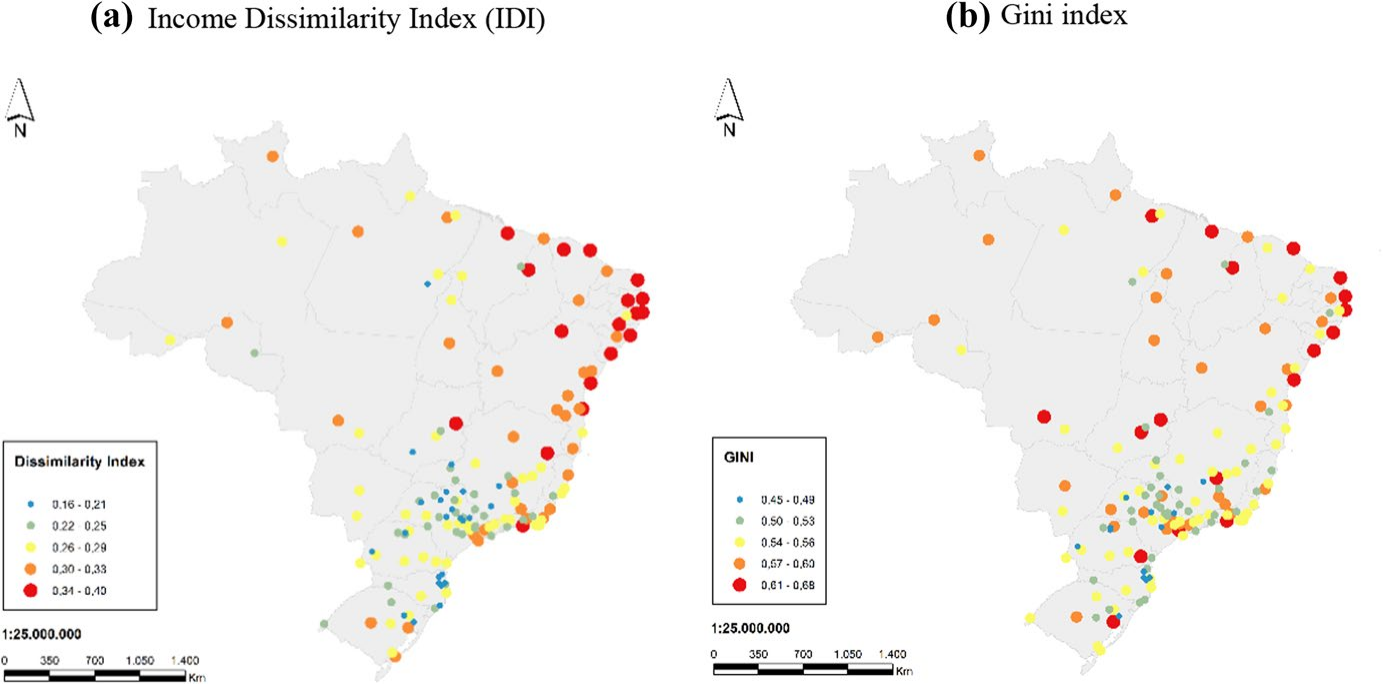
Income dissimilarity and Gini indices for 152 Brazilian cities, 2010. Source: SALURBAL Project

Income inequality and income segregation are closely related, with segregation being the spatial dimension of inequality between different social groups and an evident expression of poverty. Figure 4 shows the spatial distribution of the IDI and Gini index for the 152 Brazilian cities. Spatial patterns in income segregation are similar to spatial patterns in the Gini coefficient revealing increased income vulnerabilities in Brazil's North and Northeast regions. The North and Northeast regions are also those with the highest percentage of the country's indigenous and black population, which points to a possible correlation between income segregation and the high levels of racial inequalities in Brazil (Bailey et al. 2013).

**Fig. 5:**
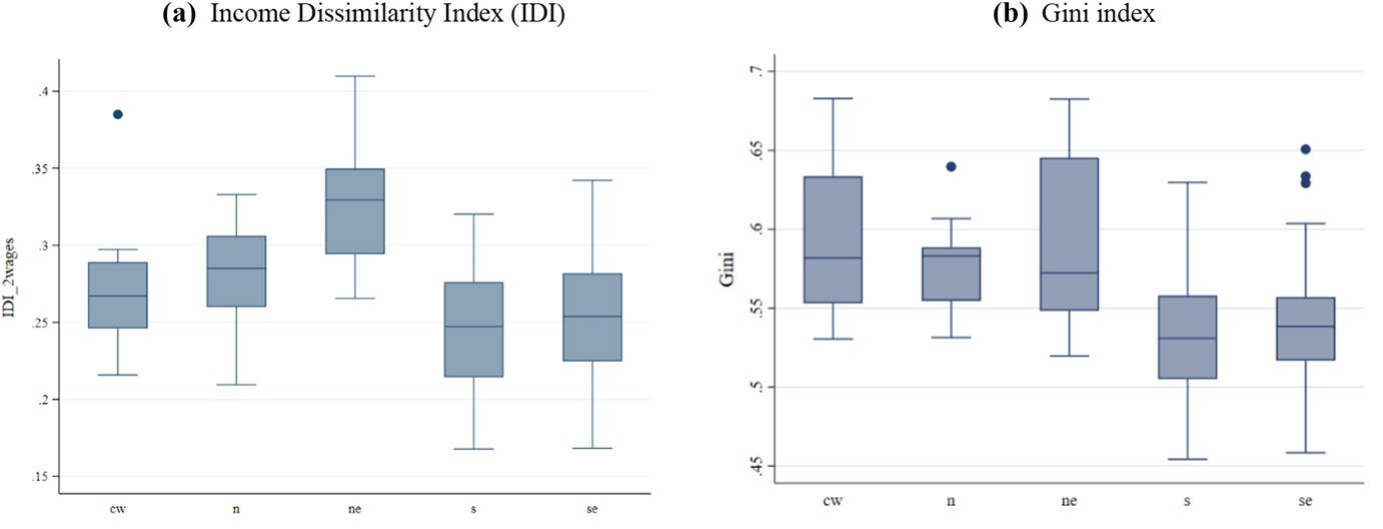
Income-based Dissimilarity and Gini indices by region. Note: Regions are abbreviated as: Midwest (cw); North (n); Northeast (ne); South (s); and Southeast (se)

Figure 5 presents the dissimilarity and Gini indices in boxplots by region. We can see that the cities in the Northeast region have the highest median income-based dissimilarity index, followed by those in the North region. Regarding the Gini index, the Midwest and North regions have the highest median, followed by the cities in the Northeast. However, the dispersion between the cities of the first quartile and the third quartile for the Northeast region is remarkable. It indicates a high level of intra-regional inequality. For the dissimilarity index and the Gini index, cities in the South and Southeast regions had lower medians but with high amplitudes.

Table 3 shows the rank of Brazil’s ten most segregated cities; nine are in the Northeast region. The exception is the city of Brasilia, also the capital of Brazil, in the Midwest region. Brasilia was planned and built between 1957 and 1960 to be the first large city of modern architecture and concentrate the Brazilian Federal public power. However, the city soon surpassed the planned borders, and settlement zones and slums soon appeared in satellite cities. The high wages of the public service also contrast with the lower income levels of the general population, making Brasília also have one of the highest inequality indices in Brazil (0.68).

**Table 3:** 10 most income segregated cities from the 152 SALURBAL sample

City	Region	State	IDI	Total population	Gini
1. João Pessoa	ne	PB	0.40	1,049,093	0.67
2. Aracaju	ne	SE	0.39	856,846	0.68
3. Brasília	cw	DF	0.38	3,235,485	0.68
4. Natal	ne	RN	0.37	1,265,118	0.64
5. Maceió	ne	AL	0.37	1,099,695	0.64
6. Teresina	ne	PI	0.36	976,798	0.63
7. Vitória de Santo Antão	ne	PE	0.35	323,316	0.55
8. Recife	ne	PE	0.35	3,588,741	0.67
9. Salvador	ne	BA	0.34	3,371,671	0.64
10. Campina Grande	ne	PB	0.34	471,572	0.58

### Associations between IDI and socioeconomic variables

**Fig. 6:**
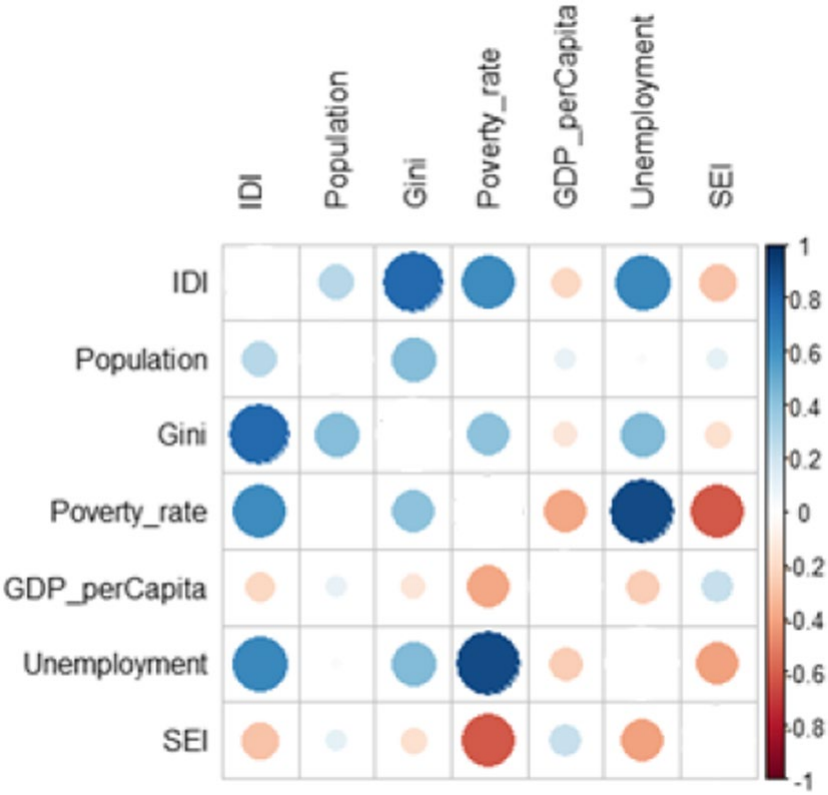
Correlation matrix among the indices. Source: Research results

Figure 6 shows the correlations between the variables. The Gini, poverty rate, and unemployment have the greatest correlations with the dissimilarity index. The total population, GDP *per capita,* and SEI have lower correlations.

Table 4 shows linear regression coefficients reflecting the associations between the dissimilarity index and the set of socioeconomic variables. We can see a strong association between some indicators with the IDI. In the first model, only the coefficient for the Gini index is presented. For this indicator, we find that with each change of 1 standard deviation in the Gini index, there is an expected increase of 0.77 (CI 95%: 0.67, 0.87) in the level of segregation.

**Table 4:** Linear regression coefficients for Brazilian cities in 2010

	Model 1 [95% CI]	Model 2 [95% CI]	Model 3 [95% CI]
Gini	0.77 (0.05) [0.67, 0.87]		0.51 (0.07) [0.37, 0.66]
Poverty rate		0.62 (0.06) [0.50, 0.75]	0.26 (0.12) [0.00, 0.51]
SEI			0.05 (0.15) [− 0.25, 0.36]
GPD *per capita*			0.00 (0.04) [− 0.09, 0.09]
Unemployment			0.18 (0.10) [− 0.02, 0.40]
Population (*log*)			0.10 (0.06) [− 0.02, 0.22]

*Standard errors in parentheses

We found a significant association between the dissimilarity index and the poverty rate in model 2. As expected, the relationship between income segregation and the indicator was positive. In other words, for each change in standard deviation in the poverty rate, there is a 0.62 (CI 95%: 0.50, 0.75) increase in income segregation. Finally, we included the entire set of variables in the last model. We observed that the Gini index and poverty rate continue to have a relevant association with residential income segregation (0.51 CI 95%: 0.37, 0.66) and (0.26 CI 95%: 0.00, 0.51), respectively. In addition to economic and social issues, we evidence that income inequality poses growing challenges given the worldwide urban changes.

We also estimated the models using the IDI corrected with the bootstrap method to provide more robustness to the results. As expected, the results were maintained. Online Appendix A shows the estimated coefficients with marginal changes, but the effects and significance remained unchanged.

Segregation is among the most prominent urban problems in rapid urbanization in developing economies. Haddad (2020) states that urbanization and urban spatial structures affect poverty and generate inequalities in social and environmental fields, further contributing to the stratification of social groups. Spatial stratification of social groups accentuates inequalities in poorer countries and is an obstacle to developing societies in the medium and long term (ECLAC 2019). Understanding how social and urban changes affect population growth, income inequality, and segregation is essential for designing policies to protect the most vulnerable people and establish viable socioeconomic development conditions for everyone.

## Challenges in the use of the dissimilarity index

The relationship between the evenness indices that measure segregation and other socioeconomic indicators is widely analyzed, especially in developed countries (Darroch 1971; Reardon and Bischoff 2011; White 1983). However, in developing economies, data on income, race, and education are more difficult to find at the census tract level, which is needed to estimate residential segregation indicators.

Around twenty indices were researched and conceptually related to some of the dimensions of residential segregation (Massey and Denton 1988). However, using an index or just one dimension of segregation does not mean excluding the others. Massey and Denton (1988) emphasize that each indicator has different distributive characteristics and that segregation is a global construction, including the five underlying dimensions of mediation, each corresponding to a different aspect of spatial variation.

Each dimension's social and behavioral implications represent a different facet of residential segregation. We refer here only to evenness, which is not measured in the absolute sense but always relative to another group. The dissimilarity index is the most common evenness indicator for calculating residential segregation and has been the mainstay of segregation research (Acevedo-Garcia et al. 2003; Massey and Denton 1988; Reardon and O’Sullivan 2004; Royuela and Vargas, 2010).

The use of census tracts to calculate the dissimilarity index is the most indicated (Fosset 2017). However, this geographic unit can also present problems. In general, the greater the degree of homogeneity within the census tract, the higher the level of apparent segregation. Moreover, the idea of homogeneity conflicts with changes in the boundaries of neighborhoods or districts, which can often happen depending on population growth. Thus, new urban areas tend to have higher levels of segregation than central areas, where there is already a predominance of defined social groups. Another limitation of using census tracts is the population density of suburban areas of cities, where housing can often be overcrowded (ECLAC 2019). Weighting problems may arise as there are different population proportions between areas.

As a spatial indicator, the dissimilarity index is sensitive to changes in geographic scale. Therefore, spatial disaggregation is correlated with the indicator's values. The Modifiable Areal Unit Problem (MAUP), also known as the scale effect, can arise (Heywood et al 1998). The problem is related to the imposition of artificial spatial units reporting on a continuous geographical phenomenon resulting in the generation of artificial spatial patterns (Royuela and Vargas 2010).

MAUP arises when aspects such as distance, the level of contact between geographic areas, and the local extent of individuals' interactions are not considered. Therefore, it is necessary to be careful when comparing levels of segregation between one city and another, especially when the size of cities is very disproportionate. According to Krupka (2007), large cities are more segregated than small ones because measures based on census tracts will tend to report higher values for the large ones, as they have more large neighborhoods to contain several census tracts. On the other hand, small cities may need to join the neighborhoods to complete a census tract.

Recent empirical research has focused on solving the upward bias problem contained in the dissimilarity index due to the growing interest in measuring residential segregation (Allen et al. 2015; Mazza 2015; Tivadar 2019). The development of tools and technologies applied to the measurement of several residential segregation indices is discussed by Tivadar (2019). According to the author, segregation remains an important issue for modern society as it has consequences for economic efficiency, social cohesion, equity, and health, among others. Therefore, despite the challenges presented regarding the dissimilarity index, the improvement of statistical tools and software reduces the uncertainties about the residential segregation indicators.

Finally, the dissimilarity index can present higher values than other indicators of segregation by income, such as the rank-order information theory index (H) presented by Garcia-López and Moreno-Monroy (2018) for 121 Brazilian cities. The H index captures the extent of residential segregation by income level. It is a weighted sum of all possible pair-wise income segregation, and its interpretation is similar to the performance given to the dissimilarity index. Thus, the H index constitutes a plausible alternative to the dissimilarity index. For the year 2010, the H index identified, as the most segregated cities, Brasília, João Pessoa, Aracaju, Maceio, Salvador and Rio de Janeiro (Garcia-López and Moreno-Monroy 2018). Therefore, this ranking has strong similarities with that offered by the dissimilarity index. Compared to results from Brazil and US, the H index has lower values for Brazilian cities than American cities, even though Brazil has higher levels of socioeconomic inequality. This evidence has also been seen in the dissimilarity index, especially regarding racial issues (Valente and Berry 2020).

Additional work examining the different types and dimensions of segregation and new measurement tools are needed to understand better how income segregation affects the social construction of individuals and families and, thereby, support the permanent reduction of urban inequality and poverty.

## Final remarks

Discussing methods of measuring segregation is not a simple matter. Segregation quantifies how heterogeneous population groups are distributed in the urban space, indicating how far an income minority group is from the city's average income. If we take a small percentage of low-income minority groups or large spatial areas as a base, the dissimilarity index can be affected by deviations from evenness, and the indicator could be strongly biased.

We present a comprehensive description of the segregation by income in the Brazilian cities as the largest urban agglomerations in the country. According to Massey and Denton (1988), a highly centralized group, spatially concentrated, unevenly distributed, clustered, and minimally exposed to the majority group, is “residentially segregated”.

To show which cut-off to define low-income households best deals with urban income segregation within large cities and the pattern of regional inequality, we use statistical analyses of correlation and distribution between the calculated IDI and the respective low-income minority group. Statistics point out that the cut-off of households earning up to 2 minimum wages yields an average proportion in the low-income minority group of 33.8% of households, seeming reasonable to work with this cut-off. Bias levels calculated on IDI using bootstrap methods were irrelevant to the study since they did not change the regressions’ estimates.

The fact that the Brazilian census tracts are built taking into account only technical criteria means that each sector's delimited area is spatially balanced, leading to each having approximately the same number of households. However, checking the indicator's bias is important for future research advancement and constitutes a new statistical framework to be taken into account when dealing with other types of residential segregation.

As expected, inequality and poverty were strongly associated with segregation. Considering that our analysis is about Brazil's most significant metropolitan areas, it is essential to highlight that this study contributes to the perspective of urban spaces characterized as socially segregated and of high spatial concentration, involving inequality and urban poverty. The advantages of urbanization have been discussed in many international forums. However, social disparities, the lack of equity in income distribution, and the possible benefits of urbanization still seem far from the reality of Brazilian cities. Furthermore, despite recent advances, the fight against poverty is an evident challenge for public policies, given the difficulty governments face in maintaining a minimum income policy capable of breaking the processes of social reproduction of poverty.

This paper has limitations in data and analysis, as the available information at the census level is from 2010. Relevant changes in income patterns have taken place in Brazil and worldwide in recent years. However, no other study has addressed such issues or used the analytical approach to investigate income segregation in Brazilian cities.

Therefore, we highlight that the study of income residential segregation plays a significant role in understanding the social relationships within large cities. Future research should focus on other determinants, in addition to income. Understanding segregation patterns by gender, race, and education is essential for developing effective social and health policies focusing on the long term. Therefore, building a consolidated research agenda that can provide adequate scientific support to guide policies to reduce segregation.

## Supplementary Information

Below is the link to the electronic supplementary material.

Supplementary file1 (DOCX 12 kb)

## Data Availability

The SALURBAL project welcomes queries from anyone interested in learning more about its dataset and potential access to data. To learn more about SALURBAL’s dataset, visit https://drexel.edu/lac/ or contact the project at salurbal@drexel.edu.

## Footnotes

1 In 2010, Brazil’s official monthly minimum wage was equal to R$ 510.00 or approximately US$ 289.00.
