# Supplementary material for: Inequality and income segregation in Brazilian cities: a nationwide analysis
Source: SN Soc Sci. 2022 Sep 10;2(9):191. doi: 10.1007/s43545-022-00491-9 (PMC9464061; doi:10.1007/s43545-022-00491-9)
Supplement: Supplementary file 1 — Supplementary file1 (DOCX 12 kb) [file 43545_2022_491_MOESM1_ESM.docx]

Appendix A. Results of the sensitivity analysis with the IDI corrected by bootstrap

|  | Model 1 (95% CI) | Model 2 (95% CI) | Model 3 (95% CI) |
| --- | --- | --- | --- |
| Gini | 0.72 (0.05)  [0.61, 0.83] |  | 0.51 (0.07)  [0.36, 0.66] |
| SEI |  | -0.77 (0.19)  [-1.16, -0.38] | 0.14 (0.16)  [-0.17, 0.47] |
| Poverty rate |  |  | 0.33 (0.13)  [0.06, 0.60] |
| GPD *per capita* |  |  | 0.00 (0.05)  [-0.10, 0.09] |
| Unemployment |  |  | 0.17 (0.11)  [-0.05, 0.39] |
| Population (*log*) |  |  | 0.01 (0.06)  [-0.11, 0.15] |

*Standard errors in parentheses.
